# Altered accumulation of osa-miR171b contributes to rice stripe virus infection by regulating disease symptoms

**DOI:** 10.1093/jxb/erx230

**Published:** 2017-07-24

**Authors:** Aizi Tong, Quan Yuan, Shu Wang, Jiejun Peng, Yuwen Lu, Hongying Zheng, Lin Lin, Hairu Chen, Yifu Gong, Jianping Chen, Fei Yan

**Affiliations:** 1 The State Key Laboratory Breeding Base for Sustainable Control of Pest and Disease, Key Laboratory of Biotechnology in Plant Protection of MOA of China and Zhejiang Province, Institute of Virology and Biotechnology, Zhejiang Academy of Agricultural Sciences, Hangzhou, China; 2 College of Plant Protection, Yunnan Agricultural University, Kunming, China; 3 School of Marine Sciences, Ningbo University, Key Laboratory of Applied Marine Biotechnology, Ministry of Education, Ningbo, China; 4 College of Agriculture and Biotechnology, Zhejiang University, Hangzhou, China

**Keywords:** *Early heading date* genes, *Ehd*, *Hd3a*, heading, miR171, *SCARECROW-LIKE6*, SCL6, symptoms, reproductive growth, rice stripe virus, vegetative growth

## Abstract

Viral infection affects the pattern of plant miRNA expression. It has been presumed that reduction of miR171 and several other miRNAs influences viral symptoms in plants. We here experimentally demonstrate the association of osa-miR171b with rice stripe virus (RSV) symptoms in rice. Inhibition of osa-miR171b caused stunting with reduced chlorophyll content in leaves similar to viral symptoms. Overexpression of osa-miR171b by an artificial miRNA extended vegetative growth and enhanced chlorophyll accumulation in leaves. Tillers were thicker, and panicles were longer with more spikelets in plants overexpressing osa-miR171b than in controls, but there were no differences in tiller numbers. Targets of osa-miR171b, *OsSCL6-IIa*, *OsSCL6-IIb*, and *OsSCL6-IIc*, were respectively up- and down-regulated in plants where osa-miR171b was inhibited or overexpressed. In plants overexpressing osa-miR171b, five positive regulators for heading development, *Ehd1*, *Ehd2*, *Ehd3*, *Ehd4*, and *Hd3a* were up-regulated, while the negative regulator *Ghd7* was down-regulated. Plants overexpressing osa-miR171b were less susceptible to RSV and virus symptoms were attenuated. Taken together, the results reveal that a reduction of osa-miR171b in RSV-infected rice contributes to RSV symptoms, and provide more insight into the roles of osa-miR171b in rice.

## Teaser

Reduction of osa-miR171b contributes to rice stripe virus symptoms by regulating its targets, while overexpression attenuates symptoms, providing direct experimental evidence that a plant miRNA affects the development of virus symptoms.

## Introduction

Rice stripe virus (RSV) belongs to the genus *Tenuivirus* and is transmitted by the small brown planthopper (SBPH; *Laodelphax striatellus* Fallén), causing serious epidemics in East Asia, including China, Japan, and Korea (Wang *et al.*, 2008). RSV has four single-stranded RNA segments. RNA1 (~9 kb) has a single open reading frame (ORF) in its complementary strand. The other three segments (RNA2, 3.5 kb; RNA3, 2.5 kb; RNA4, 2.2 kb) have ambisense polarity; each has two non-overlapping ORFs on opposite strands, separated by a non-coding intergenic region (IR) that functions in termination of transcription (Zhu *et al.*, 1991, 1992; Hamamatsu *et al.*, 1993; Takahashi *et al.*, 1993; Qu *et al.*, 1997; Wu *et al.*, 2013). RSV infection typically induces yellow stripes on leaves and considerable stunting of the plants (Iida, 1969). If infection occurs at an early stage of rice growth, the entire plant may die prematurely (Liang, 1972). Also, some leaves emerge without unfolding, then elongate and become twisted and droop (Iida, 1969).

MicroRNAs (miRNAs) are small 19–24 nt RNAs playing essential roles during the development of eukaryotes by targeting complementary mRNAs for degradation or translational repression (Carrington and Ambros, 2003; Voinnet, 2009). In plants, miRNAs regulate leaf morphogenesis, root development, flower development, and other key processes in development (Palatnik *et al.*, 2003; Chen, 2004; Guo *et al.*, 2005; Kidner and Martienssen, 2005; Wang *et al.*, 2005; Jones-Rhoades *et al.*, 2006). Research over the last decade has revealed that miRNAs also play roles in plant defense against pathogens by regulating the expression of resistance genes directly or indirectly (Navarro *et al.*, 2006; Simón-Mateo and García, 2006; Lu *et al.*, 2008; Padmanabhan *et al.*, 2009; Li *et al.*, 2011; Shivaprasad *et al.*, 2012).

Viral infection affects the pattern of miRNA expression in plants (Naqvi *et al.*, 2010; Amin *et al.*, 2011; Gao *et al.*, 2013*a*; Yin *et al.*, 2013; Feng *et al.*, 2014; Xu *et al.*, 2014). Infection by tobamoviruses, potyviruses or potexviruses altered the accumulation of particular miRNAs in *Nicotiana tabacum* (Bazzini *et al.*, 2007) and symptom severity appeared to be related to alterations in the levels of miR156, 160, 164, 166, 169, and 171 (Bazzini *et al.*, 2007). In experiments using different cucumber mosaic virus (CMV) strains, the accumulation levels of six miRNA species were significantly enhanced following infection with the severe strain CMV-Fny but less so when infected by the mild strain CMV-LS (Cillo *et al.*, 2009). Co-infection of *Nicotiana benthamiana* with potato virus X (PVX) and either potato virus Y (PVY) or plum pox virus (PPV) results in more severe systemic symptoms than in single infections. This synergism between two unrelated plant viruses is associated with, and perhaps caused by, increased accumulation of miR156, 171, 398, and 168 (Pacheco *et al.*, 2012). In kenaf (*Hibiscus cannabinus* L.) infected with hibiscus chlorotic ringspot virus, miR171 and miR168 and their targets SCL1 and AGO1 showed greater fluctuations (Gao *et al.*, 2013*a*). Both miRNAs reached the highest expression levels at about 10 d post-infection (dpi) and dropped until 30 dpi (Gao *et al.*, 2013*a*). In addition to altering the expression level of miRNAs, some viruses can induce the production of novel miRNAs with unknown functions, suggesting that miRNAs may have complex roles in virus–plant interactions (Tagami *et al.*, 2007; Guo *et al.*, 2012; Pradhan *et al.*, 2015; Zhou *et al.*, 2016).

Viruses overcome the host plant RNA silencing defense system by encoding a suppressor of RNA silencing (SRS). Many reports show that the expression or activity of host miRNAs is affected by a viral SRS (Mallory *et al.*, 2002; Kasschau *et al.*, 2003; Chapman *et al.*, 2004; Chellappan *et al.*, 2005; Simón-Mateo and García, 2006; Yu *et al.*, 2006; Csorba *et al.*, 2007; Goto *et al.*, 2007). The helper component proteinase (HC-Pro) is the SRS of potyviruses and expression of HC-Pro in Arabidopsis greatly enhances the accumulation of host miRNAs (Mallory *et al.*, 2002). Moreover, the HC-Pro of turnip mosaic virus interferes with the activity of miR171 by inhibiting miR171-guided nucleolytic function (Kasschau *et al.*, 2003). To determine whether miRNA interference is a general property of SRSs, Chapman *et al.* analysed the effect of the beet yellows virus p21, tomato bushy stunt virus p19, turnip crinkle virus coat protein, and CMV 2b silencing suppressors on miRNAs. The p21 and p19 inhibited miRNA-guided cleavage of target mRNAs, and both interacted with miRNA–miRNA* complexes *in vivo* (Chapman *et al.*, 2004). Plants expressing 2b were indistinguishable from vector-transformed plants but with modest reductions in aerial tissue weight, leaf area, and rosette diameter (Chapman *et al.*, 2004). It was later shown that 2b could weakly bind to an miR171 duplex (Goto *et al.*, 2007). AC4 protein, the SRS from African cassava mosaic virus, down-regulated host miRNA with an up-regulation of target mRNA level in transgenic Arabidopsis (Chellappan *et al.*, 2005). Further evidence showed that AC4 of African cassava mosaic virus has the ability to bind single-stranded forms of miRNAs, indicating that it blocks the normal miRNA-mediated regulation of target mRNAs presumably by inactivating mature miRNAs (Chellappan *et al.*, 2005). In these studies, plants expressing SRSs from different viruses often have deficient development. It has been suggested that the changed host miRNA pattern caused by SRSs contributes to the viral symptoms.

Although the changes in miRNA expression levels seem to correlate with viral symptoms, there has so far been little direct experimental evidence to demonstrate the association of a particular miRNA with viral symptoms. Here we demonstrate that a reduction in miR171b contributes to the viral stunting and yellowing symptoms on RSV-infected rice plants.

## Materials and methods

### Constructs

The target mimic (TM) technique has proved to be a useful tool for loss-of-function analysis of miRNAs (Franco-Zorrilla *et al.*, 2007; Todesco *et al.*, 2010; Yan *et al.*, 2012*b*). The target mimic is a sequence that is designed to bind a specific miRNA by complementation but which cannot itself be cleaved by the miRNA, hence blocking miRNA function (Franco-Zorrilla *et al.*, 2007). An improved TM technique with the ability to degrade the miRNAs by a small RNA-degrading nuclease-dependent pathway has also been developed (Yan *et al.*, 2012*b*). Here, the published TM method was used for inhibiting osa-miR171b activity with minor improvement (Supplementary Fig. S1 at *JXB* online). Briefly, a target mimic was designed to contain ten sites for binding osa-miR171b and was synthesized by Sangon Biotech (Shanghai, China) (Supplementary Fig. S1). At the binding site, the sequence is complementary to rice miR171b, but with ATCT inserted between the 12th and 13th nucleotides, so that the TM binds osa-miR171b, but cannot be cleaved by it. The synthesized TM sequence (MIM171b) was cloned into the multicloning sites of binary vector pCAMBIA1300 to construct the vector Ubi::MIM171b. The construct was verified by sequencing before being introduced into *Agrobacterium tumefaciens* strain EHA105 for transformation.

osa-miR171b was expressed in rice using an osa-MIR528 precursor-based artificial miRNA (amiRNA) (Warthmann *et al.*, 2008; Yan *et al.*, 2012*a*). This should exclude the potential effect of sequences from other parts of the osa-MIR171b precursor. To construct the amiRNA, the osa-miR528 sequence was replaced in the precursor with osa-miR171b using two primers and then cloned into the multicloning sites of pCAMBIA1300 to construct the vector Ubi::amiR171b for expressing osa-miR171b (amiR171b) (Supplementary Fig. S2). The construct was introduced into *Agrobacterium tumefaciens* strain EHA105 for transformation.

### Transformation of rice plants

Ubi::amiR171b, Ubi::MIM171b, and the empty vector were introduced into *Agrobacterium tumefaciens* strain EHA105 and then transformed into rice embryonic calli. Transgenic calli were selected on half-strength Murashige and Skoog medium containing 75 mg l^−1^ hygromycin (Sigma-Aldrich, St Louis, MO, USA). The hygromycin-resistant transgenic seedlings were then transplanted into soil and grown in the greenhouse at 28 °C.

### qPCR analysis

Total RNAs were prepared from the samples using Trizol (Invitrogen, Carlsbad, CA, USA) according to the manufacturer’s instructions. Approximately 30 µg of DNase-treated total RNA was used to prepare cDNA using the RNA PCR AMV kit (Promega, Madison, WI, USA). Oligo (dT)_18_ primer was used for PCR amplification in a reaction volume of 20 µl.

To detect osa-miR171b, the specific stem-loop RT primer was used in the reverse transcription of purified total RNA. The RT product was then used for PCR with the specific forward primer and the universal reverse primer. For SYBR green-based real-time PCR analysis, the reactions were incubated in a 384-well plate at 95 °C for 10 min, followed by 40 cycles of 95 °C for 15 s and 60 °C for 1 min. *Actin* was used as the internal control for normalization. Samples from at least three independent replicates were used for analysis, and all reactions were run in triplicate. Results were analysed by the ΔΔ*C*_T_ method. The primers used are listed in Supplementary Table S1.

### Northern blot analysis

Total RNA was isolated from frozen plant samples with Trizol (Invitrogen) according to the manufacturer’s instructions. Fifty micrograms of DNase-treated total RNA was separated on a 15% polyacrylamide gel, and transferred electrophoretically to Hybond-N+ membranes using 20× SSC. Membranes were baked at 80 °C for 2 h. DNA oligonucleotides complementary to the putative miRNA sequences were end-labeled with DIG using the DIG Oligonucleotide 3′-end labeling Kit. Membranes were pre-hybridized for at least 1 h and hybridized overnight at 42 °C using the DIG High Prime Labeling and Detection Starter Kit II. The hybridization signals were visualized by exposure to X-ray film.

### 5′ RACE

To determine where osa-miR171b cleaves its targets, amiR171b and each of the cloned targets were co-expressed in *N. benthamiana* leaves by *Agrobacterium* infiltration and at 4 dpi, total RNAs were extracted from leaves and the cDNA was reverse transcribed using the SUPERSWITCH^TM^ RACE cDNA Synthesis Kit (Sonicebiotech, Hangzhou, China) as described earlier (Shi *et al.*, 2016).

### Measurement of chlorophyll content

Leaves were collected from three replicate plants for analysis. Chlorophylls *a* and *b* were extracted with 100% methanol, and their concentrations were determined (Lichtenthaler and Wellburn, 1983).

### Inoculation of RSV

RSV was inoculated on plants as described earlier (Yan *et al.*, 2010). Viruliferous adult SBPH were transferred onto healthy rice seedlings (*Oryza sativa* L. japonica. cv. Nipponbare) and transgenic rice at the three-leaf stage. Control seedlings were inoculated with non-viruliferous planthoppers. After 72 h, the planthoppers were removed. Systemic infection was confirmed and quantitative levels of RSV RNAs were determined by real-time PCR using the primers listed in Supplementary Table S1.

## Results

### osa-miR171b was reduced in RSV-infected rice plants at 30 d post-infection

**Fig. 1.**
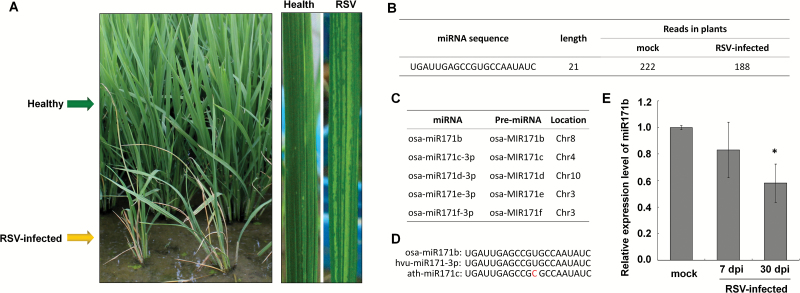
Down-regulation of an miRNA with the same sequence as osa-miR171b in RSV-infected plants showing stunting and leaf-yellow stripe symptoms. (A) Symptoms of RSV-infected rice in the field. The infected plants (front row in the image) are stunted compared with the healthy plants (back row), and have typical yellow stripes on the leaves (right panel). (B) In the small RNA library of RSV-infected plants, the reads of an miRNA with the same sequence as osa-miR171b was reduced. The number was normalized reads of the sequence ‘UGAUUGAGCCGUGCCAAUAUC’ to 1 million with the clean sequence. (C) osa-miR171b has the same mature sequence as osa-miR171c-3p, osa-miR171d-3p, osa-miR171e-3p, and osa-miR171f-3p, but they localize on different chromosomes. (D) Sequence of osa-miR171b is the same as that of *Hordeum vulgare* miR171b (hvu-miR171b) and has only one mismatched nucleotide to that of Arabidopsis miR171b (ath-miR171b). (E) Quantitative real-time PCR confirmed the reduction of osa-miR171b in RSV-infected rice plants. Samples from at least three independent rice plants infected by RSV for 30 d and with fully developed viral symptoms were used for analysis. A two-sample unequal variance directional *t*-test was used to test the significance of the difference (*P<0.05).

RSV infection causes general stunting and typical yellow stripes on rice leaves (Fig. 1A). The height of RSV-infected plants was smaller than that of non-infected plants (Fig. 1A). In previous reports, we constructed two small RNA libraries from rice infected with RSV 7 d previously and from non-infected rice using the Illumina Solexa sequencing system (Yan *et al.*, 2010; Guo *et al.*, 2012). Comparing the two libraries, it appears that the expression of osa-miR171b (UGAUUGAGCCGUGCCAAUAUC) was reduced slightly in RSV-infected rice (Fig. 1B). The sequence of osa-miR171b is the same as the mature sequence of osa-miR171c-3p, osa-miR171d-3p, osa-miR171e-3p, and osa-miR171f-3p (Supplementary Fig. S3) although they are produced from different precursors localized on different chromosomes (Fig. 1C). We here name the sequence as osa-miR171b representing the five mature miRNAs. The sequence of osa-miR171b is the same as that of *Hordeum vulgare* miR171b (hvu-miR171b) and has only one mismatched nucleotide to Arabidopsis miR171b (ath-miR171b), indicating that miR171b is conserved among plant species (Fig. 1D).

To further confirm the reduction of osa-miR171b in response to RSV infection, we used real-time PCR to compare its expression in leaves of RSV-infected rice plants at the early stages of infection (7 d post-infection, dpi) with that at 30 dpi when the symptoms were fully developed. Expression of osa-miR171b was reduced slightly at 7 dpi, but its reduction became obvious (60% of normal level) at 30 dpi (Fig. 1E).

### osa-miR171b-inhibited rice plants were stunted and had less chlorophyll in their leaves

**Fig. 2.**
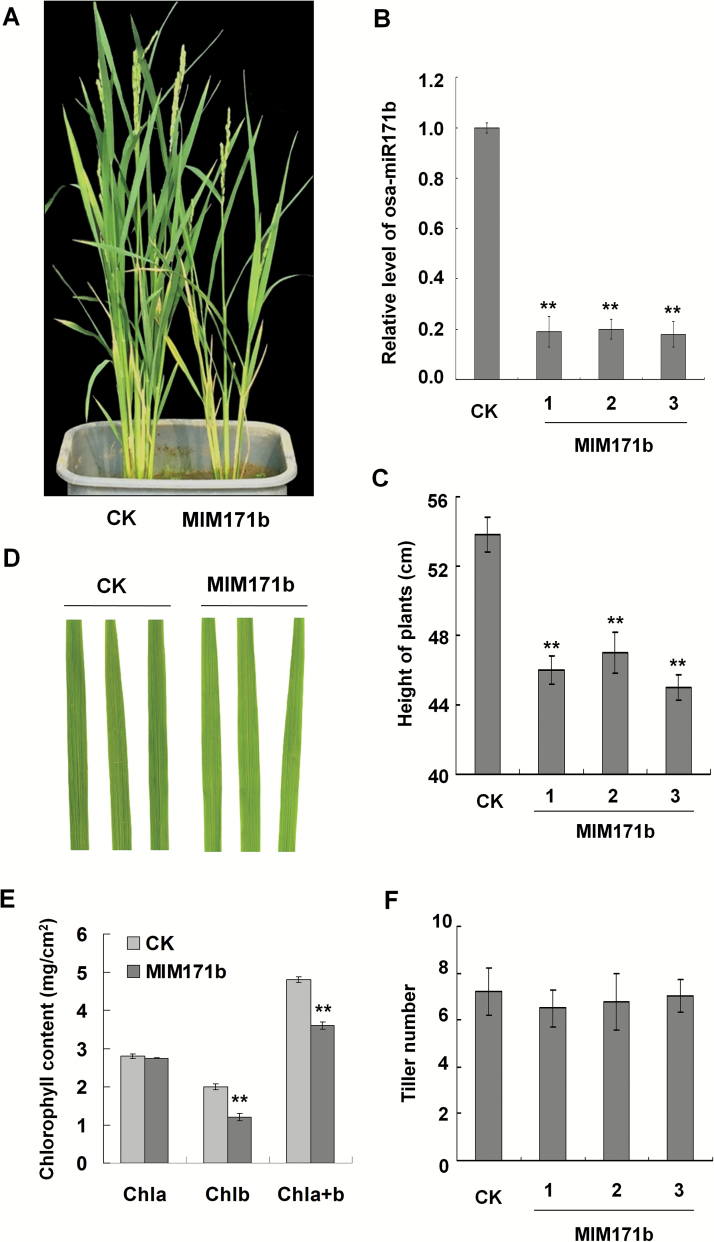
osa-miR171b-inhibited rice plants showed a stunting phenotype with reduced accumulation of chlorophyll in their leaves. (A) Stunting of osa-miR171b-inhibited rice plants (MIM171b). Plants transformed with empty vector were used as controls (CK). (B) Quantitative real-time PCR confirmed the reduction of osa-miR171b in three independent lines of MIM171b plants. Three independent rice plants of each line were used for analysis. (C) The height of MIM171b plants (three independent lines) was decreased compared with CK plants. (D) Leaves on MIM171b plants became light green. (E) MIM171b leaf contents of chlorophyll *b* and total chlorophyll *a* and *b* (*a*+*b*), were reduced but chlorophyll *a* was not affected. (F) The tiller number of MIM171b plants was similar to the control. A two-sample unequal variance directional *t*-test was used to test the significance of the difference (**P<0.01).

To explore the biological effect of osa-miR171b reduction on rice plants, we used the improved TM technique to suppress osa-miR171b in rice plants (Supplementary Fig. S1). In leaves of the regenerated osa-miR171b-inhibited (MIM171b) plants, the expression level of osa-miR171b was decreased to 25% of that in control plants transformed with the empty vector, and their height was *ca* 65% of the controls (Fig. 2A–C). The stunting phenotype of MIM171b plants was comparable to that caused by RSV infection. Moreover, leaves on MIM171b plants appeared light green (Fig. 2D) and their chlorophyll *b* (or total chlorophyll *a* and *b*) content was reduced (Fig. 2D, E). There was no obvious difference in number of tillers between MIM171b and control plants (Fig. 2F).

### Overexpression of osa-miR171b resulted in extended vegetative growth

To examine the direct effects of osa-miR171b expression, osa-miR171b was overexpressed in rice plants through an artificial miRNA (amiRNA) method based on the osa-MIR528 precursor (Supplementary Fig. S2). This method was chosen rather than transforming the osa-MIR171b precursor because it excludes the potential effect of other sequences from the osa-MIR171b precursor. An artificial osa-MIR528 precursor containing an unrelated amiRNA was used as a control.

**Fig. 3.**
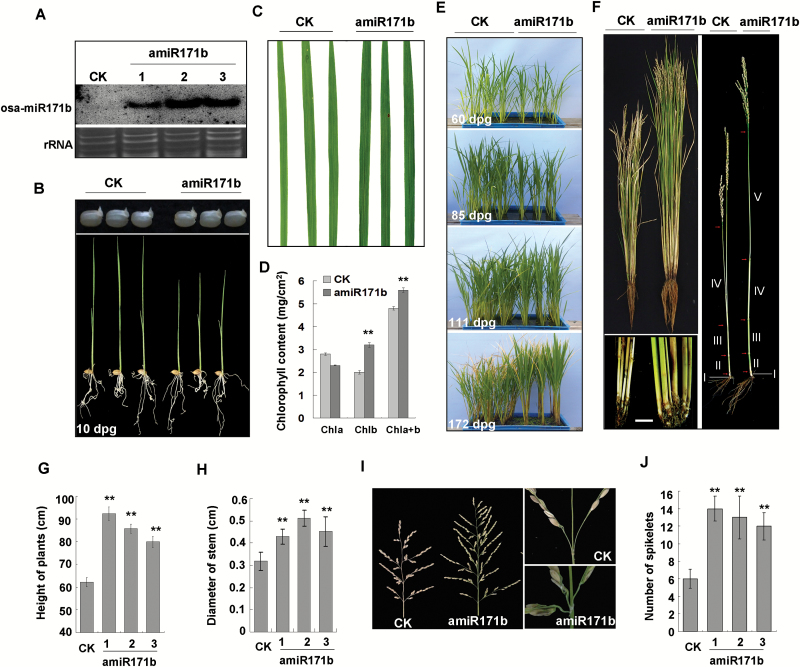
Phenotype of rice plants overexpressing osa-miR171b (amiR171b) by the artificial miRNA method. (A) The overexpressed osa-miR171b accumulated at a high level in three independent lines of transgenic plants. (B) Seeds of amiR171b plants and control plants germinated simultaneously (upper lane) but 10 d post-germination (dpg) plantlets of amiR171b were shorter than the control. (C) Leaves of amiR171b appeared dark green. (D) The contents of chlorophyll *b* and total chlorophyll *a* and *b* (*a*+*b*) were increased in amiR171b leaves, while the content of chlorophyll *a* was decreased slightly. (E) amiR171b plants were shorter than the control at 60 d, while at 85 d when the control plants began to head and stopped extending, amiR171b plants kept growing and finally became taller than control plants at 111 d. At 172 d, the spike of amiR171b plants had expanded completely, and the plants were significantly taller than control plants. (F) The mature amiR171b plants were significantly higher than the controls (left panel). There were four nodes and four internodes (I–IV) on stems of control plants, but five nodes and five internodes (I–V) on amiR171b plants (right panel). The tillers of amiR171b plants were also much thicker than control plants (bottom left panel). Bar represents 1 cm. (G) The height of amiR171b plants (three independent lines) was significantly greater than control plants. (H) Diameter of amiR171b tillers (from three independent lines) was significantly larger than control. (I) The panicle of amiR171b plants was much longer with more spikelets (left panel). Notably, from some panicle nodes of amiR171b plants three spikes (upper right panel) were produced instead of the two (bottom right panel) from control panicle nodes. (J) More spikelets were present on amiR171b panicles (from three independent lines). A two-sample unequal variance directional *t*-test was used to test the significance of the difference (**P<0.01).

Sequences of osa-miR171b accumulated at a high level in three independent lines of transgenic plants expressing amiR171b (named amiR171b plants) (Fig. 3A). Seeds of amiR171b plants germinated at the same time as those from control plants (Fig. 3B) but the seedlings grew more slowly and had dark green leaves with a lower content of chlorophyll *a*, but an additional 50% of chlorophyll *b* (and an additional 20% of chlorophyll *a*+*b*) (Fig. 3C, D). Eighty-five days after germination, the control plants began to head and were taller than the amiR171b plants (Fig. 3E, F). The control plants did not grow further but the amiR171b plants continued to grow and at 111 d, when they started heading, they were taller than the controls. At 172 d, the spikes of amiR171b plants had expanded completely, and the plants were significantly higher than the controls (Fig. 3E, G) with an additional node on their stems (Fig. 3F, G). There was no significant difference in tiller numbers between amiR171b and control plants (data not shown) but tillers of amiR171b plants were significantly thicker (Fig. 3F, H). The panicles of amiR171b plants were also bigger with more spikelets (Fig. 3I, J) and three spikes were produced from some panicle nodes instead of the two on control plants (Fig. 3I).

### Expression of heading-related genes in amiR171b and MIM171b plants

**Fig. 4.**
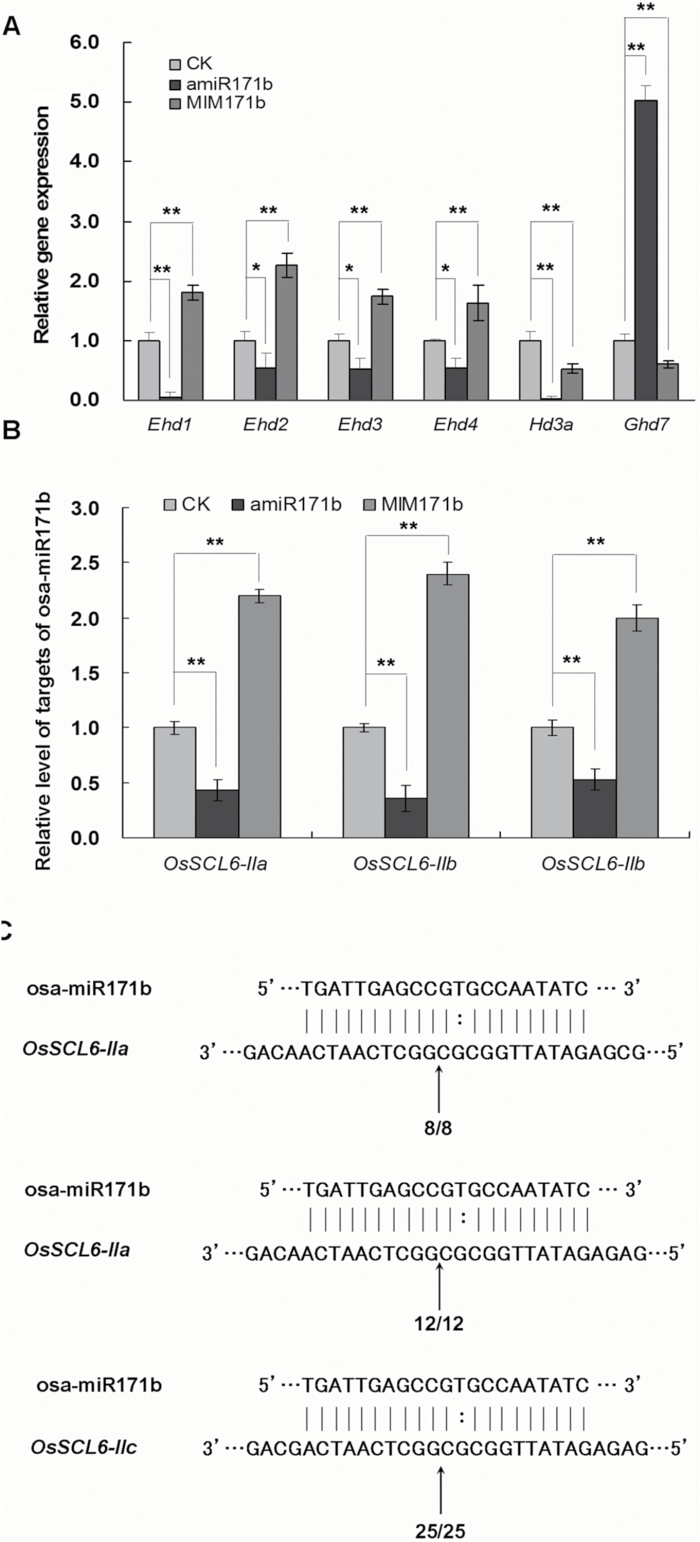
Expression of heading-related genes and the potential targets of osa-miR171b in amiR171b and MIM171b plants. (A) The positive regulators of heading, *Ehd1*, *Ehd2*, *Ehd3*, *Ehd4*, and *Hd3a*, were extremely down-regulated, while the negative regulator, *Ghd7*, was up-regulated in amiR171b plants. By contrast, in MIM171b plants, *Ehd1*, *Ehd2*, *Ehd3*, and *Ehd4* were up-regulated, and *Ghd7* was down-regulated. All of the expression changes were significant (*P<0.05; **P<0.01). (B) The targets of osa-miR171b, *OsSCL6-IIa*, *OsSCL6-IIb*, and *OsSCL6-IIc*, were up-regulated in MIM171b plants, but significantly down-regulated in amiR171b plants (**P<0.01). In quantitative real-time PCR, three independent plants of each kind were used for analysis. (C) Result of 5′-RACE showed the potential cleavage site (arrow) of osa-miR171b on three targets, *OsSCL6-IIa*, *OsSCL6-IIb*, and *OsSCL6-IIc*. Clone frequency is indicated by the number.

Heading was substantially delayed in amiR171b plants (Fig. 3F). Four *Early heading date* (*Ehd*) genes are known to play important roles in regulating rice heading. *Ehd1* promotes heading in rice by up-regulating the expression of *Heading date 3a* (*Hd3a*) (Doi *et al.*, 2004; Takahashi *et al.*, 2009). *Ehd2* promotes heading by up-regulating *Ehd1* (Matsubara *et al.*, 2008). *Ehd3* normally functions as a negative regulator of *Ghd7*, which is a repressor of *Ehd1* in floral induction (Matsubara *et al.*, 2011). *Ehd4* up-regulates the expression of the ‘florigen’ gene *Hd3a* through *Ehd1* (Gao *et al.*, 2013*a*). Examination of the expression of these heading-related genes in leaves of amiR171b plants showed that the positive regulators of heading, *Ehd1* and *Hd3a*, were extremely down-regulated, and *Ehd2*, *Ehd3*, and *Ehd4* were reduced by around 50%, while the negative regulator of heading, *Ghd7*, was up-regulated substantially in amiR171b plants (Fig. 4A). Thus *Ehd1*, *Hd3a*, and *Ghd7* were affected drastically by overexpression of osa-miR171b, while their upstream regulation genes, *Ehd2*, *Ehd3*, and *Ehd4*, were less affected.

In leaves of MIM171b plants, *Ehd1*, *Ehd2*, *Ehd3*, and *Ehd4* were up-regulated, while *Ghd7* was down-regulated, which is consistent with results from amiR171b plants, and indicates a possible connection between osa-miR171b and heading-related genes. Meanwhile, the expression of *Hd3a*, a gene downstream of *Ehd1*, was not up-regulated in MIM171b plants, which may explain why the heading of MIM171b plants did not occur earlier than in control plants, and suggests that there may be other pathways participating in the regulation of *Hd3a* in MIM171b plants (Fig. 4A).

### Expression levels of osa-miR171b targets in amiR171b and MIM171b plants

It has been reported that GRAS gene family members *SCARECROW-LIKE6-II* (*SCL6-II*), *SCL6-III*, and *SCL6-IV* genes are the targets of ath-miR171c in Arabidopsis (At) (Wang *et al.*, 2010). To identify the targets of osa-miR171b and examine their expression in rice, we BLAST searched the homologues of *AtSCL6-II*, *AtSCL6-III*, and *AtSCL6-IV* genes in rice. Three sequences were identified. Multiple alignment and phylogenetic analysis showed that the amino acids of all of them were most closely related to AtSCL6-II (Supplementary Fig. S4A, B) and we therefore named them *OsSCL6-IIa* (Accession No.: XM_015771585), *OsSCL6-IIb* (Accession No.: XM_015771929) and *OsSCL6-IIc* (Accession No.: XM_015781657). The binding sites to osa-miR171b on the three sequences were identical (Supplementary Fig. S4C).

To determine whether these genes were regulated by osa-miR171b, we first investigated their expression in leaves of amiR171b and MIM171b plants. All three genes were up-regulated in MIM171b, but down-regulated in amiR171b plants suggesting that they could be targeted by osa-miR171b (Fig. 4B). We next determined the cleavage site of osa-miR171b on the targets in a transient system by 5′RACE analysis. All targets were cleaved at the position between the 10th and 11th nucleotide in the binding sites of osa-miR171b, which further demonstrates the targeting of osa-miR171b to the three genes (Fig. 4C).

We also predicted other potential targets of osa-miR171b using psRNATarget (http://plantgrn.noble.org/psRNATarget /?function=1). In addition to the three sequences already identified, eight sequences were predicted but there were no obvious expression changes in any of these in amiR171b and MIM171b plants (data not shown) and we do not therefore consider them to be cleavable targets of osa-miR171b.

### RSV symptoms on amiR171b plants were attenuated

**Fig. 5.**
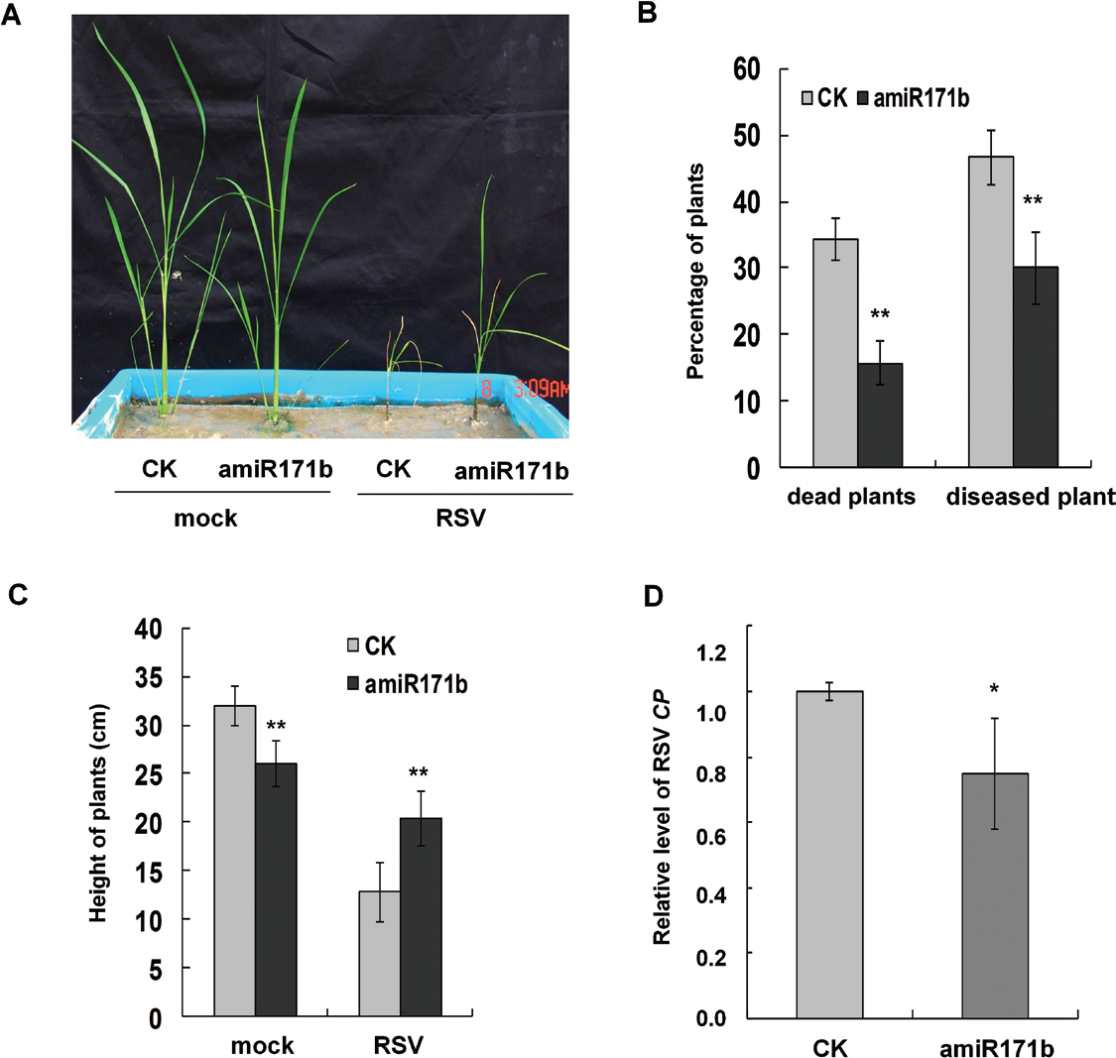
RSV symptoms on amiR171b plants were attenuated and disease incidence was lower. (A) Typical symptoms of RSV on amiR171b and control plants. (B) At 30 d post-inoculation (dpi), significantly fewer amiR171b plants had viral symptoms (diseased) or were dead than control plants. (C) The surviving RSV-infected amiR171b plants were taller than RSV-infected control plants, indicating the attenuation of viral symptoms on amiR171b plants. (D) RSV coat protein (CP) accumulated less in amiR171b plants than in controls. A two-sample unequal variance directional *t*-test was used to test the significance of the difference (*P<0.05; **P<0.01).

We finally investigated the response of amiR171b plants when challenged with RSV. RSV was inoculated to plants at the two-leaf stage using the method described earlier (Yan *et al.*, 2010). At 30 dpi, 34.3% of control plants were dead and 46.7% showed the typical viral symptoms (Fig. 5A, B) whereas on amiR171b plants only 16.5% had died and 30.0% had symptoms (Fig. 5A, B). Among the symptomatic plants, those overexpressing osa-miR171b were significantly taller than the controls, and contained lower levels of RSV RNAs, indicating that overexpression of osa-miR171b attenuated the symptoms of RSV, and conferred some resistance to RSV (Fig. 5C, D).

## Discussion

Several viruses have been reported to reduce miR171 in infected plants (Bazzini *et al.*, 2007; Naqvi *et al.*, 2008; Cillo *et al.*, 2009; Pacheco *et al.*, 2012; Gao *et al.*, 2013*b*) and we here showed the reduced expression of osa-miR171b in RSV-infected rice plants at 30 dpi. Two papers have reported the changed expression of rice miRNAs following RSV infection (Lian *et al.*, 2016; Yang *et al.*, 2016). Yang *et al.* used stringent criteria to identify the differentially expressed miRNAs: miRNAs that had more than 100 reads were selected for analysis, while miRNAs with reads less than 100 were not considered. osa-miR171s, osa-miR171b, osa-miR171c, osa-miR171d, and osa-miR171e were all expressed at a low level and therefore the changed expression of osa-miR171b was not analysed in the paper (Yang *et al.*, 2016). Lian *et al.* analysed the time course response of rice miRNAs to RSV infection. Results showed that osa-miR171s was up-regulated soon after infection (3 dpi), but was at normal levels or lower at 15 dpi, indicating a reduction of osa-miR171s during the later stages of infection (Lian *et al.*, 2016). Our present results show a further reduction of osa-miR171s in rice at 30 dpi. Similar fluctuations in expression of miR171 in response to virus infection were also observed on hibiscus chlorotic ringspot virus-infected kenaf where miR171 and miR168 were both up-regulated at the early stages of infection, reached the highest expression levels at about 10 dpi, but dropped to 50% of normal levels at 30 dpi (Gao *et al.*, 2013*b*). It will be interesting to investigate whether such fluctuation of miR171 generally happens with other viruses.

SRSs are the main pathogenic factor. Their expression in plants leads to a change in many miRNAs. Several SRSs affect expression and function of miR171. HC-Pro of turnip mosaic virus inhibits miR171-guided nucleolytic function, interfering with the function of miR171 (Kasschau *et al.*, 2003). Tomato bushy stunt virus p19 binds miR171 duplex efficiently, while CMV 2b binds weakly to an miR171 duplex (Goto *et al.*, 2007). In our previous work, we produced transgenic rice plants expressing RSV p3 protein, the SRS of RSV, and these had no obvious deficiency in development (Wu *et al.*, 2014). The results indicate that RSV SRS probably does not directly affect osa-miR171b. Recently, it was reported that levels of the unprocessed miRNA precursors pre-miR156 and pre-miR171 increased in sweet orange plants infected by citrus psorosis virus, while their mature miRNAs were decreased significantly (Reyes *et al.*, 2016). Further results showed that the citrus psorosis virus 24K protein could interact with pre-miR156a or pre-miR171a, which suggests that a viral protein may alter host miRNA patterns by affecting the processing of an miRNA precursor. At present, we have no idea whether such regulation of osa-miR171b exists in the interaction between RSV and plants.

It has been presumed that the reduction in miR171 and several other miRNAs contributes to viral symptoms (Bazzini *et al.*, 2007; Pacheco *et al.*, 2012; Gao *et al.*, 2013*b*). We have now examined experimentally the contribution of a reduction of osa-miR171b to RSV symptoms. MIM171b plants were stunted and had less chlorophyll in their leaves (Fig. 2), while amiRN171b plants kept growing for a longer time and had a higher content of chlorophyll in their leaves. Moreover, there were fewer dead or diseased amiR171b plants after RSV infection and their height was greater than that of the diseased controls. All these results support the conclusion that a reduction in osa-miR171b contributes to the stunting and yellowing symptoms following RSV infection of rice.

In our previous report, we identified many chloroplast-related genes (ChRGs) down-regulated in RSV-infected *N. benthamiana*. Of the 11 down-regulated genes whose silencing caused plant chlorosis, nine were ChRGs, indicating that down-regulation of ChRGs contributes to the viral chlorosis or yellowing symptom. Here, MIM171b plants had the expected up-regulated expression of *OsSCL6-IIa*, *OsSCL6-IIb*, and *OsSCL6-IIc* and had significantly less chlorophyll *b* and chlorophyll *a*+*b* in their leaves, which is consistent with the results from Arabidopsis (Fig. 2D, E). In Arabidopsis, At*SCL6-II*, *AtSCL6-III*, and *AtSCL6-IV* are regulated by ath-miR171c and themselves negatively regulate chlorophyll biosynthesis (Wang *et al.*, 2010; Ma *et al.*, 2014). Moreover, amiR171b plants had significantly increased amounts of chlorophyll *b* and chlorophyll *a*+*b* (Fig. 3C, D). Both results indicate that osa-miR171b resembles ath-miR171c in regulating chlorophyll synthesis by targeting *OsSCL6-IIa*, *OsSCL6-IIb*, and *OsSCL6-IIc*. Hence, the yellowing symptoms of RSV appear to be related to the reduction of osa-miR171b, as well as the down-regulation of ChRGs.

One phenotype of MIM171b plants is stunting. As there have been no reports on the effects of inhibited miR171 expression in other plants, we do not know whether this is a general effect. We did not examine the effects of overexpressing osa-miR171b targets, and there are no published results of such studies, so we cannot exclude the possibility that the stunting phenotype is also regulated by osa-miR171b by targeting *OsSCL6-IIa*, *OsSCL6-IIb*, and *OsSCL6-IIc*.

The attenuated symptoms of RSV on amiR171b plants further confirms the role of osa-miR171b in the development of RSV symptoms (Fig. 5) and suggests that amiR171b plants have an improved resistance to RSV (Fig. 5). This effect may be related to the accumulation of chlorophyll. Chloroplasts play an important role not only in capturing the energy from sunlight but also in pathogen defense by stimulating the hypersensitive response and systemic acquired resistance, as well as synthesizing the precursor of jasmonate, an important defense molecule (Padmanabhan and Dinesh-Kumar, 2010). Many chloroplast-related genes have also been identified that confer resistance to particular viruses. An h-type thioredoxin, functioning in photosynthesis, was reported also to participate in the resistance of tobacco to tobacco mosaic virus and CMV (Sun *et al.*, 2010). Rubisco and Rubisco activase also defend against tobamoviruses (Bhat *et al.*, 2013; Zhao *et al.*, 2013). Here, the increased accumulation of chlorophyll in amiR171b plants may strengthen the defense function of chloroplasts, hence increasing resistance to RSV. We are not sure whether the overexpressed osa-miR171b could interfere with the function of RSV SRS, hence making amiR171b plants resistant to RSV. The present evidence does not exclude this possibility, which is worth investigating next.

The miRNA171 family is an ancient miRNA family that is conserved in many plant species (Llave *et al.*, 2002). In Arabidopsis, plants overexpressing miR171c and *scl6-II scl6-III scl6-IV* triple mutant plants have smaller shoots, and the triple mutant plants additionally exhibit pleiotropic phenotypes, including an increased chlorophyll accumulation, decreased primary root elongation, and abnormal leaf and flower development (Wang *et al.*, 2010). In barley, overexpression of miR171 causes a branching defect, extends the vegetative phase, increases the number of short vegetative phytomers, and delays the differentiation of spikelet meristems into floral organs (Curaba *et al.*, 2013). Recently, Fan *et al.* (2015) identified a rice T-DNA insertion mutant where the expression of OsMIR171c is up-regulated. The mutant had a prolonged vegetative phase, delayed heading date, and bigger shoot apex, which is largely consistent with the results from barley (Fan *et al.*, 2015). Taken together, these results show that miR171 plays a conservative role in regulating meristem identity, but its effect on the phase transition may be monocot-specific. Our results support a regulatory function for miR171 on phase transition in rice plants (Fig. 3). amiR171b plants had phenotypes consistent with those reported for rice by Fan *et al.* (2015), including a longer phase of vegetative growth and increased final height, but there were some differences. For example, we did not observe any significant difference in tiller numbers between amiR171b and control plants, there was only one additional node on amiR171b plants, and the thicker tillers and longer panicles we found were not reported for plants overexpressing OsMIR171c. These minor differences may be due to the different method used for overexpressing osa-miR171. By using the artificial miRNA technique to express osa-miR171b, we would have excluded any effects of miRNA* and other phased miRNAs possibly produced from the precursor of osa-miR171b.

In plants, *Ehd* genes play important roles in heading regulation. *Ehd1* promotes heading in rice by positively regulating the expression of *Hd3a* (Doi *et al.*, 2004; Takahashi *et al.*, 2009). *Ehd2* promotes heading by up-regulating *Ehd1* (Matsubara *et al.*, 2008). *Ehd3* normally functions as a down-regulator of *Ghd7*, which is a repressor of *Ehd1* in floral induction (Matsubara *et al.*, 2011). *Ehd4* up-regulates the expression of the ‘florigen’ genes *Hd3a* through *Ehd1* (Gao *et al.*, 2013*a*). In rice plants overexpressing osa-MIR171c, expression of the positive regulator *Hd3a* was up-regulated (Fan *et al.*, 2015). We also found that *Hd3a* was up-regulated in our amiR171b plants and also that four other positive regulators, *Ehd1*, *Ehd2*, *Ehd3*, and *Ehd4*, were all up-regulated, while the negative regulator, *Ghd7*, was down-regulated. Moreover, their expressions were changed in the opposite way in MIM171b plants. These results indicate that these genes or the heading development were regulated by osa-miR171b but it is not known whether such regulation was executed by the targets of osa-miR171b.

In summary, our results demonstrate a reduction of osa-miR171b in RSV-infected rice, especially at the later stages of infection, and show that such reduction contributes to RSV symptoms. The study did not determine which target(s) of osa-miR171b are responsible for such an effect, the detailed connection between osa-miR171b and regulation of heading, or the mechanism by which expression of osa-miR171b is down-regulated by RSV infection. Further studies are needed to examine these questions.

## Supplementary data

Supplementary data are available at *JXB* online.

Fig. S1. Construction of target mimic sequence of osa-miR171b.

Fig. S2. Construction of artificial miRNA for expressing osa-miR171b.

Fig. S3. Alignment of members in THE osa-miR171 family.

Fig. S4. Identification of targets of osa-miR171b.

Table S1. Primers used for analysis.

## Supplementary Material

Supplmentary_Figures_S1_S4_Table_S1

## Abbreviations:

amiR171b: artificial osa-miR171b
At: *Arabidopsis thaliana*
CMV: cucumber mosaic virus
dpi: days post-infection
Ehd: Early heading date
HC-Pro: Helper component-proteinase
MIM171b: target mimic sequence of osa-miR171b
miRNA: microRNA
Os: *Oryza sativa*
PPV: plum pox virus
PVX: potato virus X
PVY: potato virus Y
RSV: rice stripe virus
SBPH: small brown planthopper
SCL6-II: SCARECROW-LIKE6-II
SRS: suppressor of RNA silencing
TM: target mimic.
