# Supplementary material for: Altered accumulation of osa-miR171b contributes to rice stripe virus infection by regulating disease symptoms
Source: J Exp Bot. 2017 Jul 24;68(15):4357–67. doi: 10.1093/jxb/erx230 (PMC5853540; doi:10.1093/jxb/erx230)
Supplement: Supplmentary_Figures_S1_S4_Table_S1 [file erx230_suppl_supplmentary_figures_s1_s4_table_s1.pdf]

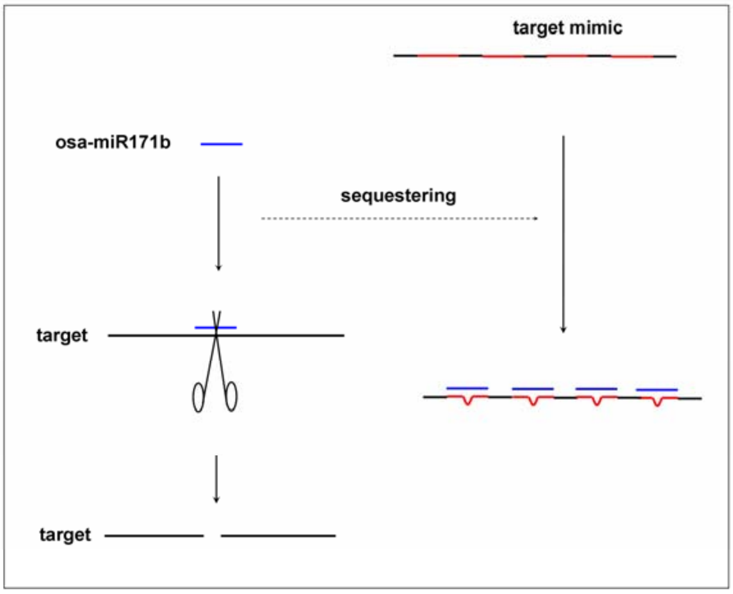

Sequence of target mimic:

GAGCTCGATATTGGCACATCTGCTCAATCAAGCTAGCTGATATTGGCACATCTGCTCAATCA
AGCTAGCTGATATTGGCACATCTGCTCAATCAAGCTAGCTGATATTGGCACATCTGCTCAAT
CAAGCTAGCTGATATTGGCACATCTGCTCAATCAAGCTAGCTGATATTGGCACATCTGCTCA
ATCAAGCTAGCTGATATTGGCACATCTGCTCAATCAAGCTAGCTGATATTGGCACATCTGCT
CAATCAAGCTAGCTGATATTGGCACATCTGCTCAATCAAGCTAGCTGATATTGGCACATCTG
CTCAATCAGGATCC

Suppl. Fig. S1 Construction of target mimic sequence of osa-miR171b. A. The strategy for inhibiting miRNA activity by target mimic of miRNAs. The target mimic is designed to contain ten copies of the binding site (red line) that can bind the specific miRNA (blue line), but cannot be cleaved. When expressed in plants, it can sequester miRNA, hence preventing miRNAs from binding their normal silencing targets. B. The synthesized target mimic sequence for inhibiting osa-miR171b.

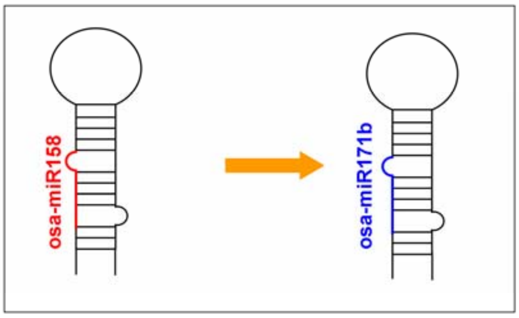

Sequence of amiR171:

GAGCTCTTTGGCTGTAGCAGCAGCAG**TGATTGAGC**
CGTGCCAATATCCAGGAGATTCAGTTTGAAGCTGG
ACTTCACTTTTGCCTCTCTGATATTGGGGCGGTTCA
ATCATTCCTGCTGCTAGGCTGTTCGGATCC

Suppl. Fig. S2 Construction of artificial miRNA for expressing osa-miR171b. A. The method for constructing the artificial osa-miR171b in the osa-MIR528 precursor. B. The sequence of the constructed amiR171.

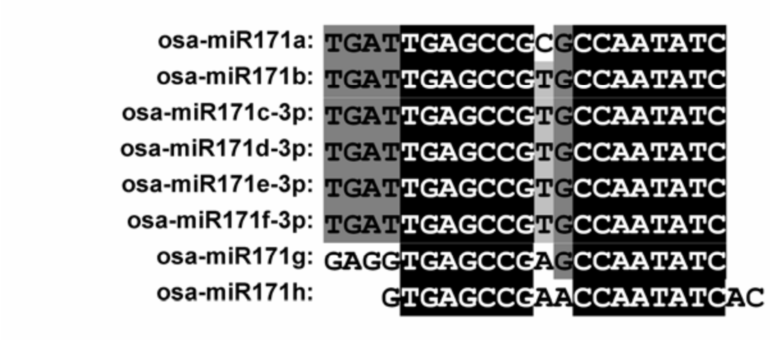

Suppl. Fig. S3 Alignment of members of the osa-miR171 family.

A

	AtSCL6-II	AtSCL6-III	AtSCL6-IV
OsSCL6-IIa	28.79%	24.41%	27.01%
OsSCL6-IIb	28.49%	26.84%	26.72%
OsSCL6-IIc	27.55%	26.84%	27.06%

B

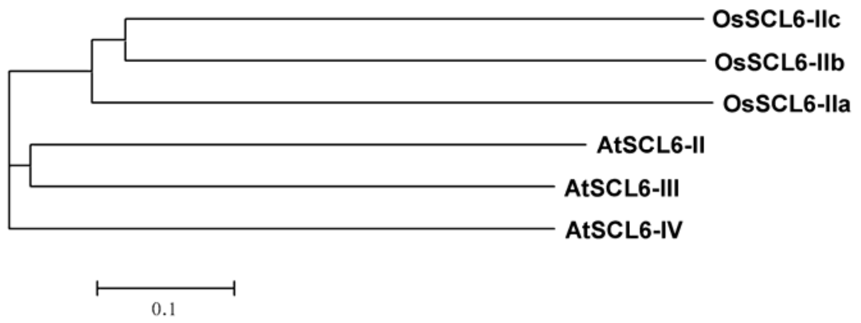

C

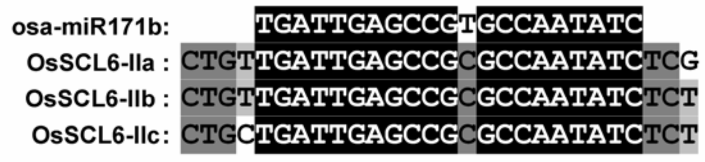

Suppl. Fig. S4 Identification of targets of osa-miR171b. A. Three sequences were identified with higher amino acid identity to *AtSCL6-II* than to *AtSCL6-III* and *AtSCL6-IV*. We therefore named them *OsSCL6-IIa*, *OsSCL6-IIb* and *OsSCL6-IIc*. B. Phylogenetic tree constructed with *AtSCL6-II*, *AtSCL6-III*, *AtSCL6-IV*, *OsSCL6-IIa*, *OsSCL6-IIb* and *OsSCL6-IIc*. C. The binding sites of osa-miR171b on *OsSCL6-IIa*, *OsSCL6-IIb* and *OsSCL6-IIc* (their reverse complementary sequences were used for alignment).

Suppl. Table S1 Primers used for analysis

Primers Name	sequence	Usage
miR171b-RT	5'-GTTGGCTCTGGTGCAGGGTCCGAGGT ATTCGCACCAGAGCCAACGATATT -3'	RT for osa-miR171b
miR171b-F	5'-GCGTCTGTGATTGAGCCGTGCC-3'	q-PCR for osa-miR171b
microq-R	5'-CTGGTGCAGGGTCCGAGGTA-3'	q-PCR for osa-miR171b
OsSCL6-IIa-F	5'-ATCTACCTCGCCCGTAACCA-3'	q-PCR for OsSCL6-IIa
OsSCL6-IIa-R	5'-AAGAAGGAAGCCCACTGACC-3'	q-PCR for OsSCL6-IIa
OsSCL6-IIb-F	5'-CGCCAGGTCTCGCCTATTC-3'	q-PCR for OsSCL6-IIb
OsSCL6-IIb-R	5'-AGCCGTGAGCTTCAACAACG-3'	q-PCR for OsSCL6-IIb
OsSCL6-IIc-F	5'-ACTCCGTGCCCGACAAT-3'	q-PCR for OsSCL6-IIc
OsSCL6-IIc-R	5'-CGCCTGAGTTACCGTGAAG -3'	q-PCR for OsSCL6-IIc
Ehd1ReF	5'-GCGCTTTTGATTTCCTGC-3'	q-PCR for Ehd1
Ehd1ReR	5'-ATATGTGCTGCCAAATGTTGCT-3'	q-PCR for Ehd1
Ehd2ReF	5'-GGAAGAGGGTGTACGTGTGC-3'	q-PCR for Ehd2
Ehd2ReR	5'-AGCCTTGCACTCTCTTCAGC-3'	q-PCR for Ehd2
Ehd3ReF	5'-GACAGGTCTAGCGAGCAACC-3'	q-PCR for Ehd3
Ehd3ReR	5'-GGCACAGTTCGTTTCGGTAT-3'	q-PCR for Ehd3
Ehd4ReF	5'-CAGGTGTTTGCACAGATTGG-3'	q-PCR for Ehd4
Ehd4ReR	5'-ACGCCTCCTCTGAAGTAGCA-3'	q-PCR for Ehd4
Hd3aReF	5'-GCTCACTATCATCATCCAGCATG-3'	q-PCR for Hd3a
Hd3aReR	5'-CCTTGCTCAGCTATTTAATTGCATAA-3'	q-PCR for Hd3a
Ghd7ReF	5'-AGGTGCTACGAGAAGCAAATCC-3'	q-PCR for Ghd7
Ghd7ReR	5'-GGGCCTCATCTCGGCATAG-3'	q-PCR for Ghd7
OsActin-F	5'-GTATCCATGAGACTACATACAACT-3'	internal control for q-PCR
OsActin-R	5'-TACTCAGCCTTGGCAATCCACA-3'	internal control for q-PCR
RSVCP-F	5'-ATGCAAGACGTACAAAGGACAG-3'	RT-PCR for RSV
RSVCP-R	5'-CTATGTTTTGTGTAGAAGAGGTT-3'	RT-PCR for RSV
